# Fecal microbiota transplantation in metabolic syndrome: History, present and future

**DOI:** 10.1080/19490976.2017.1293224

**Published:** 2017-02-27

**Authors:** P. F. de Groot, M. N. Frissen, N. C. de Clercq, M. Nieuwdorp

**Affiliations:** a Department of Internal and Vascular Medicine, Academic Medical Center, Amsterdam, The Netherlands; b Wallenberg Laboratory, Department of Molecular and Clinical Medicine, Sahlgrenska Academy, University of Gothenburg, Gothenburg, Sweden; c Department of Internal medicine, VU University Medical Center, Amsterdam, The Netherlands; d ICAR, VU University Medical Center, Amsterdam, The Netherlands

**Keywords:** microbiota, fecal transplantation, metabolic syndrome

## Abstract

The history of fecal microbiota transplantation (FMT) dates back even to ancient China. Recently, scientific studies have been looking into FMT as a promising treatment of various diseases, while in the process teaching us about the interaction between the human host and its resident microbial communities. Current research focuses mainly on *Clostridium difficile* infections, however interest is rising in other areas such as inflammatory bowel disease (IBD) and the metabolic syndrome.

With regard to the latter, the intestinal microbiota might be causally related to the progression of insulin resistance and diabetes. FMT in metabolic syndrome has proven to be an intriguing method to study the role of the gut microbiota and open the way to new therapies by dissecting in whom insulin resistance is driven by microbiota. In this article we review the history of FMT, the present evidence on its role in the pathophysiology of metabolic syndrome and its efficacy, limitations and future prospects.

## The history of fecal microbiota transplantation

The recent scientific upsurge in the field of gut microbiota has firmly established its role in contemporary clinical medicine. However, the history of fecal infusions in medicine is much longer. Today we know that fecal microbiota transplantation (FMT) or ‘bacteriotherapy’ may also transfer host phenotype. This section will provide a historical overview of FMT indications and applications, and how it has evolved into its current use.

### Historical applications of fecal transplantation (300 AD –1950 AD)

The first records of fecal transplantation date back to 4th century China, where “yellow soup” was applied in cases of severe food poisoning and diarrhea.^1^ Subsequent records reverently speak of “golden syrup.”^2^ By the 16th century, the Chinese had developed a variety of feces-derived products for gastrointestinal complaints as well as systemic symptoms such as fever and pain.^1^ Meanwhile, Bedouin groups were said to have consumed the stools of their camels as a remedy for bacterial dysentery.^3^ Italian anatomist and surgeon Acquapendente (1537–1619) further extended this to a concept he coined “transfaunation,” the transfer of gastrointestinal content from a healthy to a sick animal, which has since been applied extensively in the field of veterinary medicine.^4^ Interestingly, many animal species are found to naturally practice coprophagia, leading to a greater diversity of microorganisms in their intestines, enabling them to digest a greater number of food sources.^5^

Slowly these ideas began to spark interest in 18th century European physicians. German born Christian Paullini (1643–1712) was the first to outline the therapeutic potential of human excretions in his work the “Heilsame Dreck-Apotheke” (literally: healing mud pharmacy).^4^ The fundamental discovery by Antoni van Leeuwenhoek that his stool contained microbes – “God's smallest creatures”^6^ – as well as observations from the Russian zoologist Metchnikoff (1845–1916), laid an early foundation for the modern field of microbiota study.^7^ Inspired by the reports of longevity in Bulgarian farmers despite their poor living conditions, Metchnikoff introduced fermented products in his diet and noted improvements in his general health. He hypothesized this to be due to an altered balance in colonic microbes, with an increase in lactic acid bacteria (still called “*Lactobacillus bulgaricus*”) protecting against senescence-accelerating toxins. Metchnikoff's bacteria captured public interest and were successfully marketed during his lifetime. His concept of increasing the number of beneficial microbes in the gut in an attempt to improve human health, clearly shows a historic use of probiotics *avant la lettre*. In a similar light, German bacteriologist and physician Alfred Nissle isolated an *Escherichia coli* strain that to date bears his name. Initially, the microorganism was found protective against *Shigella* outgrowth and subsequent gastroenteritis, but its impact in human health was later extended to include chronic inflammatory conditions.^8^

Soon gut bacteria would also be found valuable in recovering from infectious gastroenteritis. When German soldiers of the ‘Afrikakorps’ were dying of locally contracted dysentery in the early 1940s, Nazi scientists were determined to find a cause and cure. Observations of better-faring locals who would take in fresh camel stools upon the first signs of illness led them to analyze the feces and isolate *Bacillus subtilis*. Subsequent culturing and administration of the bacterium resolved the disease in many.^9^

By now, the numerous examples of microbes influencing human health have set the stage for a more thorough examination of its applications, and the first controlled studies using therapeutic fecal suspensions have emerged.

### FMT in modern science (1950s-2000)

**Figure 1.**
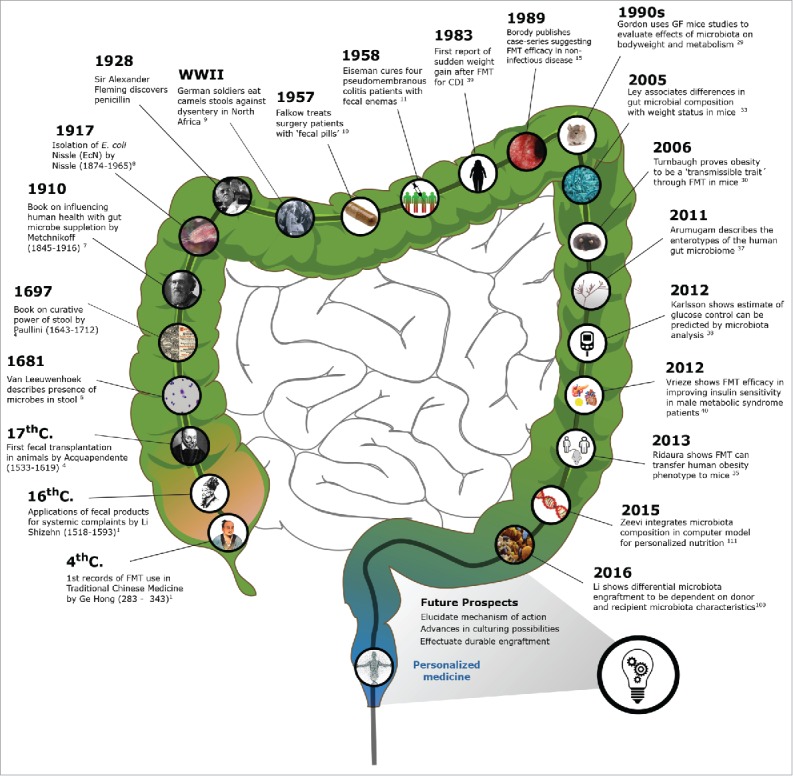
Timeline: Key contributions to FMT development and research.

Antibiotics, since their discovery generously prescribed, have ended an era in which infectious diseases were the most common cause of death. But antibiotics also came with side-effects and antimicrobial resistance. In an attempt to ameliorate collateral damage on commensal microbes, bacteriologist Stanley Falkow sampled fecal material from surgical patients before starting them on pre-procedural antibiotics (Fig. 1).^10^ After converting the stools into pill form, he prescribed their daily intake to half of the group during post-surgical recovery, an idea so repelling at the time it got him fired when the administrative board found out. Anecdotal evidence from this study in the early 1950s describes better outcomes in the treatment group, but the data of the unofficial ‘Ersatz’^10^ trial were never published.

In the succeeding year, a group of Colorado-based surgeons led by Dr. Eiseman performed an experiment along the same rationale: that restoration of a healthy gut microbial balance can improve patients' health. After numerous other therapies had failed to effectuate recovery, they treated 4 patients critically ill with pseudomembranous colitis with fecal enemas from healthy donors. Results were impressive, with rapid and complete recovery in all subjects.^11^ In the following 2 decades, 16 more cases were selected to undergo the same procedure. The attained 94% success rate despite the poor prognosis in this therapy-refractory patient group proved promising.^12^

By this time, the cause of this potentially life-threatening condition had been identified as infection by the gram-positive anaerobe spore forming bacterium *Clostridium difficile*, often provoked by antibiotic use.^13^ Because of clinical similarities between infectious and non-infectious types of colitis, physicians started to speculate whether FMT could also be of help in inflammatory bowel disease and irritable bowel syndrome (IBD and IBS).^14^ The earliest record of FMT for a non-infectious disease concerns a 45-year old male having refractory ulcerative colitis (UC), showing full and lasting clinical recovery upon “an exchange of bowel flora.”^15^ Numerous subsequent case studies focused on a multitude of gastrointestinal complaints.^15-17^ In the majority of cases, patients were found to “[positively] respond to manipulation”^17^ of their microbiome following the colonic administration of a mixture of microflora from unaffected individuals.

By the turn of the century, new insights connecting gut microbes to the pathophysiology of extra-intestinal diseases would broaden the applications of FMT.

### FMT in extra-intestinal disease (2000-current)

Over 700 patients are reported to have been treated with FMT for recurrent *Clostridium difficile* infection (CDI) since Eiseman's experiment.^4^ Three recent randomized controlled trials have reported cure rates of 90% or higher.^18-20^ In general, the treatment effect is lasting,^21,22^ and safe, with no related side-effects or newly acquired medical conditions during follow-up, even when performed in vulnerable patient groups.^18,21,23-25^

As the burden shifted from infectious to non-communicable disorders, the range of FMT applications extended. Recent case-reports even include incidental findings of post-FMT remission in extra-gastrointestinal conditions like multiple sclerosis,^14^ Parkinson,^26^ idiopathic thrombocytopenic purpura^14^ and chronic fatigue.^14^ Another microbiota-associated disease is kwashiorkor (severe malnutrition), which Smith and colleagues studied in 2013 in children in Malawi. They investigated 317 twin pairs for 3 y (from birth until 3 y of age). During these 3 years, 50% of the twin pairs stayed well nourished, but 7% manifested concordance for acute malnutrition and 43% became discordant for malnutrition.^27^ Hereafter, they transplanted fecal microbiota from discordant pairs into germ-free mice. Mice that received gut microbiota from kwashiorkor children showed significant weight loss, along with dysregulation of carbohydrate and amino acid metabolism.^28^

Strategies attempting to tackle the other end of the metabolic spectrum, the obesity epidemic, have failed to generate satisfactory results.^29,30^ However, much recent animal and human evidence points toward a possible role for gut microbiome manipulation in reestablishing energy homeostasis. The following section will briefly review how the microbiome has become connected to the pathophysiology of metabolic syndrome and how FMT may aid future therapy.

### Animal FMT studies in metabolic syndrome

Experimental evidence in animals connecting the intestinal microbiota to the metabolic syndrome is widely available. Owing to the creation of germ-free (GF) mice by prof. Jeffrey Gordon and technological advances in sequencing methods, scientists were able to prove causality of microbial involvement in weight management and glucose and lipid metabolism.

For example, Bäckhed and colleagues induced weight gain and increased insulin resistance in GF (C56BL/6) mice upon oral administration of fecal material from their conventional counterparts, despite a simultaneous reduction of food intake.^31^ Researchers attributed this to a more effective carbohydrate uptake (and subsequent lipolysis leading to increased body fat content) due to processing of nutrients by the microorganisms present.^31^ Conventionalization of GF mice also amplified the weight gain after putting these mice on a Western or high-fat diet (HFD).^29,32^ Next, the recipient GF mice of feces from obese (ob/ob) donor mice put on significantly more weight compared with recipients of feces from lean donor mice.^30^

Ley et al. (2005) went on to show that under similar dietary circumstances, ob/ob mice carry significantly less Bacteroidetes and more Firmicutes in their guts compared with lean ob/+ and wild-type mice.^33^

A recent study with Sprague-Dawley rats confirmed this microbial signature, where fecal transplants from rats on a control diet restored a rise in plasma fatty acid and glucose intolerance after fructose-induced metabolic syndrome.^34^ Interestingly, glucose tolerance improved significantly through the administration of an antibiotic mix of ampicillin and neomycin. Ridaura and colleagues (2013) were the first to transplant human feces into GF (C57BL/6J) mice and confirmed increased weight gain upon transfer of fecal material derived from an obese adult compared with that of her lean twin.^35^ Moreover, co-housing the obese sample recipients with lean-donor animals showed a decrease of obese phenotype acquisition.

Finally, a recent study by Liou et al. (2013) suggests that the positive metabolic effects of Roux-en-Y gastric bypass (RYGB) surgery is partly be due to an altered composition of the gut microbiota.^36^ They have performed FMT in GF-mice from patients that had undergone RYGB surgery. This procedure resulted in significant loss of weight and fat mass compared with the GF-mice that had received gut microbiota from mice that had undergone a sham procedure. Data validating these effects in humans are eagerly awaited.

### FMT in human metabolic syndrome

In the same period, further emphasis was placed on the role of gut microbiota in metabolic disease in humans and on human microbiota variation. Of note, Arumugam described different human enterotypes^37^ and Karlsson showed that analysis gut microbiota composition can predict metabolic status.^38^

Interestingly, the first record of sudden weight gain following FMT dates from 1983, as an incidental finding after resolution of recurrent CDI in an adult female patient. Another such case of “new-onset obesity” was published recently, warranting caution in considering the use of obese donor material for fecal transplantations.^39^

Concerning the treatment of metabolic syndrome with FMT, the only human study to date was performed by Vrieze et al. (2012), which suggests that FMT from lean unaffected donors temporarily increases peripheral insulin sensitivity (with a similar yet not statistically significant trend toward improved hepatic insulin resistance).^40^ In line with results obtained from the animal models, these changes were found to be positively correlated with an increase in the number of butyrate-producing bacteria in the gut.

In conclusion, previous findings suggest a causal relation between the gut microbiota and metabolic syndrome, but the (patho)physiologic pathways remain to be elucidated. In the next section we will first discuss the pathophysiology of metabolic syndrome and then the role that gut microbiota may play.

### The role of gut microbiota in the pathophysiology of the metabolic syndrome

The prevalence of metabolic syndrome and its sequelae has risen to epidemic proportions to become one of the most pressing global health problems of our time. The metabolic syndrome is a cluster of symptoms, defined by insulin resistance, dyslipidemia, high blood pressure and increased abdominal girth, which are strongly associated with the development of type 2 diabetes and cardiovascular disease. The hepatic manifestation of metabolic syndrome, though not part of its criteria, is non-alcoholic fatty liver disease (NAFLD) and its vascular manifestation is atherosclerosis.

The historical overview has explained how we came to use FMT in the context of metabolic syndrome. In this section we will first address the pathophysiology of the metabolic syndrome in general with the role of low-grade inflammatory changes particular and then continue by focusing on the role of the gut microbiota and and the possible mechanisms through which they influence host metabolism.

### Inflammation and insulin resistance

Studies in the early 20^th^ century already showed that high-dose salicylates attenuate glycosuria in patients, providing early clues that insulin resistance is secondary to inflammation. This was largely ignored until the 1990s, when it was shown that insulin resistance can be provoked by pro-inflammatory cytokines such as tumor necrosis factor α (TNFα).^41^ A review by Shoelson et al. in 2006 provides an excellent summary on the inflammatory mechanisms of insulin resistance.^42^ One important pathway is through c-Jun amino-terminal kinases (JNK), activated by inflammatory cytokines and free fatty acids. JNK is increased in obese individuals and absence of JNK results in improved insulin sensitivity.^43^ Inflammatory pathways (such as JNK and NF-κB) are also induced by obesity-related endoplasmic reticulum (ER) stress.^44^ JNK changes the function of insulin receptor substrate 1 (IRS-1), which regulates insulin and insulin-like growth factor pathways.^45^ These pathways play an important role in age-related disease and life span in animal models.^46,47^ Also, inflammation goes hand-in-hand with lipid accumulation in the vessel wall as an early sign of atherosclerosis, demonstrating the link between lipid accumulation, atherosclerosis and inflammation as hallmark of the metabolic syndrome.^42^

Another important mechanism that plays a role in insulin sensitivity and obesity is mediated through nuclear peroxisome proliferator-activated receptors (PPARs), of which PPARγ is best characterized. PPARγ is expressed in many tissues including liver, muscle and adipose tissue and its activation attenuates hyperglycemia and hyperlipidemia by regulation of metabolic genes.^48^ Thiazolidinediones, a class of synthetic oral glucose lowering drugs, are strong agonists of PPARγ. Macrophages highly express PPARγ, further intertwining inflammation and insulin resistance.^48^ In vitro studies have shown that statins also induce PPARγ-mediated transcriptional activity in macrophages, beside other anti-inflammatory effects like inhibiting lipopolysaccharide-induced TNFα transcription and the NF-κB pathway,^49^ implying the effects of statins in metabolic syndrome may in fact be partly anti-inflammatory and mediated through PPARy.

In conclusion, there is a firm base of evidence linking the spectrum of metabolic dysfunction seen in metabolic syndrome to inflammation. In the next paragraph we present evidence that this inflammation may derive from the gut.

### “Leaky gut”

In the last decades, a solid base of evidence has linked the inflammatory state in metabolic syndrome to impaired gut barrier function and leakage of bacteria and/or bacterial components into the system.^50,51^ Resulting low grade endotoxemia (and possibly bacteremia) chronically activates inflammatory pathways.^52^ Bacterial components may also migrate to target organs,^53,54^ leading to an influx of macrophages that contribute to local as well as systemic low grade inflammation and insulin resistance.^52^ The intestinal barrier consists of many components each contributing to its function in unique ways: an epithelial lining conjoined by junction proteins, thick (‘outer’ and ‘inner’) mucus layers, a bacteria deterring glycocalix,^55^ luminal immunoactive components such as IgA, cytokines and mast cell proteases and gut-associated lymphoid tissue trained to discriminate commensals from pathogens.^56^ Malfunction of any component can be described as impaired gut barrier function and may or may not lead to bacterial translocation depending on the defect. These different components of the gut barrier can be assessed in different indirect ways, each with its own limitations, as elegantly reviewed by Grootjans et al.^57^ Several clues that the gut becomes permeable to bacteria in metabolic syndrome have been uncovered, but it should be noted that direct evidence of actual live bacterial translocation in humans in the context of metabolic syndrome has not yet been delivered. The first studies linking gut barrier disruption to metabolic derangement have shown increased paracellular transport and impaired tight junction function using oral ingestion of substances, such as lactulose and mannitol, sucralose, polyethylene glycols (PEG) or ^51^Cr-EDTA and measuring their urinary excretion.^58^ It is unlikely however that large molecules, let alone bacteria, are transported paracellularly.^59^ In contrast, bacteria may enter the body by endocytosis, which has been observed in the absence of tight junctional damage,^59^ when the epithelial cell layer is compromised by apoptosis, cell damage, during physiologic cell shedding, or when the mucus layer is impaired. Incidentally, a recent study emphasized the importance of dietary fibers in maintaining the mucus layer by showing that fiber-deprived microbiota use the colonic mucus layer as an alternative food source.^60^

A different approach to demonstrate impaired intestinal barrier function is by looking for bacterial signatures in the circulation. Many diseases, including metabolic syndrome, have been linked to endotoxemia, i.e. the presence of lipopolysaccharide (LPS) in the blood, a supposed proxy for translocation of gram-negative bacteria. LPS infusion in rodents showed an increase in insulin resistance to a similar extent as a high-fat diet.^61^ However, in humans this is still not proven due to reliability issues of endotoxin assays, conflicting study outcomes and the question whether LPS found in metabolic syndrome subjects is actually bioactive.^62^ Also, transportation of endotoxin and bacteria in metabolic syndrome occurs mainly by route of the lymphatic system and the portal vein.^63,64^ In both cases, it will first pass a target organ (the liver or via the thoracic lymph duct and the arterial circulation to a different tissue), before it can be measured in a venous blood sample, complicating adequate measurement in humans.

Finally, data in various disease states support the hypothesis of microbiota translocation to the blood stream^51^ and target organs such as the vessel wall in atherosclerosis,^53^ the liver in non-alcoholic steatohepatitis^65^ and mesenteric adipose tissue in inflammatory bowel disease.^54^ It would be very interesting to investigate whether presence of gut bacteria can also be demonstrated in the blood, the mesenteric lymph nodes or other peripheral tissues of human metabolic syndrome subjects, e.g. by taking tissue samples during elective surgery.

### Ways in which the gut microbiota contribute to the metabolic syndrome

We have already described the inflammatory mechanisms through which gut microbes might affect host metabolism. Another important mechanism is the production of signaling molecules by gut microbes. These can affect gut integrity, the immune system and satiety and may impact host metabolic phenotype. Short-chain fatty acids (SCFAs) provide the most well-studied example. SCFAs, such as acetate, propionate and butyrate, are small molecules metabolized by gut microbes from dietary fibers, for which receptors are ubiquitous in the body, resulting in numerous complex effects, reviewed by Den Besten et al. in 2013^66^ and by Canfora et al.^67^ in 2015 in more detail. Their effects include upregulation of tight junction proteins such as claudin-1 (butyrate),^68^ epigenetic regulation of immune cells through HDAC-inhibition (butyrate),^69^ altering of intestinal gluconeogenesis,^70^ increasing plasma incretin hormones, reduction of TNFα (acetate)^71,66^ and changing lipid and glucose metabolism (propionate, butyrate).^72,73^

Bile acids (BA) are another class of molecules that play a role in microbiota-host communication, mediated by nuclear receptor farnesoid X receptor (FXR). This interaction seems an important determinant of metabolic health, as FXR-deficient mice that are fed a high-fat diet or those that are genetically predisposed toward obesity have better glucose regulation than control mice with normally functioning FXR.^74^ Evidence from animal studies is solid, but human studies on this topic are still lacking. Current evidence on microbiota-BA interaction and the receptors involved was well-reviewed recently by Wahlström et al.^74^

Finally, metagenomic studies in humans have identified various specific gut microbiota changes in individuals with metabolic syndrome^38^ including malevolent microbes that contribute to insulin resistance, such as *Prevotella copri* and *Bacteroides vulgatus*,^75^ but also beneficial species such as *Akkermansia municiphila*,^76,77^ and *Faecalibacterium prausnitzii*,^78^ which are associated with increased insulin sensitivity. Of note, a newly discovered mechanism of action of the widely used glucose lowering drug metformin was shown in diet-induced obese mice, where it improved glucose homeostasis by increasing the population of *Akkermansia* species.^76^ Several other examples of specific interactions of gut microbes with the host are discussed in the following paragraphs by reviewing the literature on the involvement of gut microbes in several other aspects of the metabolic syndrome: atherosclerosis, hepatic steatosis and elevated blood pressure.

### Microbiota and atherosclerosis

The connection between gut microbiota and atherosclerosis was already described in 1999, when endotoxin levels, following bacterial translocation, were found to be independently correlated with cardiovascular outcome and carotid atherosclerosis measured by duplex ultrasound.^79^ Traditional risk factors explain about half of the atherosclerotic burden in linear regression. Genetics are believed to explain another 10 percent. Microbiota and their many metabolic products may largely account for the rest.^80^ For example, DNA of oral microbiota *Veillonella* and *Streptococcus* was found in plaques of individuals with atherosclerosis and their abundance correlated with the abundance of these species in the oral cavity.^53^ As for gut microbiota, Karlsson et al. found in 2012 that atherosclerosis is associated with a different gut metagenome.^81^ One mechanism of microbiota-mediated atherosclerosis induction that has been elucidated is through L-carnitine and phosphatidylcholine (from red meat, cheese and eggs). These food components are first converted by the microbiota to TMA, then by the liver into TMAO, which increases atherosclerotic burden^82^ and promotes a prothrombotic phenotype.^83^ Microbiota can also protect from atherosclerosis, as recently shown when *Akkermansia municiphila* reversed Western diet-induced atherosclerosis and endotoxemia in ApoE-knockout mice.^77^ Another recent study in ApoE-KO mice showed that probiotic mixture VSL#3 can protect from atherosclerosis.^84^ Furthermore, germ free mice showed attenuation of vascular leukocyte infiltration and adhesion and of monocyte attractant protein MCP-1 and proinflammatory cytokine IL-17 in response to an angiotensin II challenge.^85^

### Microbiota and non-alcoholic fatty liver disease and -steatohepatitis

Several human studies show microbiota differences in people with non-alcoholic steatohepatitis (NASH)^86^ or non-alcoholic fatty liver disease (NAFLD),^87,88^ compared with healthy controls. Also, people with NALFD have increased LPS-binding peptide levels in the plasma.^89^ In individuals with NASH levels are even higher and correlate to TNF-α mRNA expression in liver tissue, pointing toward a role for endotoxemia in inflammatory liver steatosis.^89^ Furthermore, apart from increased endotoxemia, people with NAFLD have increased intestinal permeability, which correlates with bacterial overgrowth in the small intestine and severity of steatosis.^90^ No human studies on FMT in NASH or NAFLD have been performed yet, but in mice it has been shown that antibiotics can reduce portal endotoxin levels and hepatic accumulation of lipids.^91^ Furthermore, adding *Bifidobacterium pseudocatenulatum* to a high-fat diet (HFD) decreased insulin resistance and liver steatosis when compared with only a HFD in C57BL-6 mice.^92^ Also, microbiota transfer of inflammasome-deficient mice to wild-type mice exacerbated hepatic steatosis and portal endotoxin influx.^93^ In line, microbiota transfer from a mouse with a HFD-induced phenotype of insulin-resistance and inflammation reproduced this phenotype in recipient mice, whereas the microbiota from a mouse fed the similar HFD that did not develop insulin resistance or inflammation failed to do so.^94^ These studies all show microbiota-mediated effects on liver phenotype in mice. A human intervention trial, albeit not placebo controlled, showed a decrease in intrahepatic triglyceride content measured by magnetic resonance spectroscopy upon addition of probiotics to usual care.^95^ Finally, since decades, increased intestinal permeability has been linked to alcohol (ab)use.^96^ Recently however, studies point toward a role for ethanol-producing microbes in the etiology of NASH and NAFLD,^97^ bridging alcoholic and ‘non-alcoholic’ causes of steatohepatitis through the microbiome. A randomized clinical trial is currently being performed at our department to see if the effects of fecal microbiota transfer from healthy donors can attenuate NAFLD and NASH severity (Dutch trial register, http://www.trialregister.nl/NTR4339).

### Microbiota and blood pressure

Elevated blood pressure is a common feature of metabolic syndrome, but literature on gut microbial involvement is still modest. Several interesting discoveries have been made that suggest a more direct influence than merely through an increase of atherosclerotic burden. First of all, a decreased microbiota diversity and a decreased Firmicutes/Bacteroidetes ratio in humans and in 2 rat models for hypertension was found. More importantly, the blood pressure in these rat models could be corrected by increasing the Firmicutes/Bacteroidetes ratio with antibiotics.^98^

A possible mechanism through which alterations in the gut microbiota can induce hypertension is through SCFAs, as it was recently found that SCFA-receptor olfactory receptor 78 (Olfr78) is expressed in the kidney and mediates renin production in response to propionate.^99^ Moreover, this receptor and G protein-coupled receptor 41 (GRP41) were found in the smooth muscle cells of blood vessels, where they increase blood pressure in response to microbiota-derived SCFAs.^99^

### Future perspectives: Microbiota as therapy

Now that we have outlined current evidence on gut microbial involvement in the metabolic syndrome, we will speculate in this final section on the therapeutic possibilities offered by this promising new field of gut microbiota.

Although only one small placebo-controlled RCT has altered the gut microbiota through FMT and showed causality in the interaction between gut microbiota and metabolic syndrome in humans,^40^ many studies have shown that gut microbiota play a role in all aspects of the metabolic syndrome, including insulin resistance, dyslipidemia, atherosclerosis, hepatic steatosis and elevated blood pressure. Now is the time to further investigate these claims using well-designed randomized placebo-controlled studies featuring FMT that focus on unraveling the mechanisms through which gut bacteria interact with host metabolism, by monitoring microbiota changes and engraftment of beneficial and pathogenic bacterial strains in the intestinal microbiome over time,^100^ recording changes in metabolites (e.g., short-chain fatty acids, bile acids, incretin hormones), investigating genetic and epigenetic effects (e.g., on the immune system), all while taking dietary habits into account, to ultimately develop novel, more attractive and personalized strategies to manipulate the microbiota.^101^

As mentioned earlier, the most widely accepted explanation for the origin of metabolic syndrome is translocation of endotoxin (LPS) or direct translocation of gram-negative microbes, causing low grade inflammation. However, data on this phenomenon are still inconclusive. Current research should look extensively into this topic, since it is a key component that will lead to insight and hopefully effective treatment modalities in the future.

Furthermore, until recently the majority of intestinal microbes could not be cultured. However recent endeavors in culturing these mostly anaerobic microbiota proved successful and challenged the notion that most microbiota is unculturable.^102^ Browne and colleagues used fresh fecal samples to test a novel method based on targeted phenotypic culturing, using broad-range growth medium called YCFA. Overall, 137 distinct species were isolated, 90 of which were on the Human Microbiome Project's “most wanted” list of previously uncultured and unsequenced microbes.^102^ These inspiring results will hopefully lead to further insights on the function and interactions between various gut microbes and help improve understanding which cases will respond to FMT and which will not, which remains a vital question. Moreover, it is not only the question *which* microbes should be infused but also *how many* species or strains are needed to alter the gut microbiota effectively.^103^ Studies to date have mainly been limited to genus- and species-comparisons and have not clarified to which extent donor microbiota colonizes a recipient.^104^ Previous findings in non-FMT settings found that newly introduced (non-pathological) strains are not able to persevere in an established gut ecosystem, particularly if the species were already present.^105,106^ We have recently found that effective colonization by donor fecal bacteria (engraftment) is partly driven by the gut microbial composition of the recipient and differs between metabolic syndrome subjects.^104^ The next step will be a more precise manipulation of the gut microbiota, for example by introducing specific microbes to outcompete undesirable strains.

Finally, FMT has logistical challenges and is associated with several risks (e.g., infections), undesirable outcomes (e.g., increased risk of microbiota-associated diseases, such as new-onset obesity) and distaste by the recipients and doctors, the so called ‘yuck’factor.^107^ Isolating specific bacterial strains for the production of novel probiotics could be a safer and more elegant treatment.^108^ This process raises 2 major challenges that preclude rapid translation into clinical practice. First of all, regulation of FMT can be a major hurdle. The US Food and Drug Administration (FDA) has determined that FMT constitutes a biologic product and drug and therefore maintained that the FMT process (e.g., donor eligibility, screening and stool processing) falls under FDA jurisdiction.^109^ Because safety and efficacy has not yet been proven with large RCTs, investigational new drug application (IND) is required for fecal transplantation in the US. In Europe however, FMT has no such status, which makes it easier to use FMT in clinical trials while at the same time studying pathophysiological mechanisms. In this phase we deem it critical to characterize donors in detail, constantly adjust donor screening to the most recent insights and to closely monitor long-term effects in recipients. After beneficial strains have identified using FMT, another hurdle concerns the culturing and storage of therapeutically interesting bacterial strains under good manufacturing practice, which are 2 essential, but also time consuming factors.^108^

In conclusion, FMT will allow us to study the pathophysiology of metabolic syndrome and help us to identify novel therapeutic targets. In this endeavor, we should study specific host genetic, epigenetic, dietary and microbial characteristics to be able to predict therapeutic efficacy of these novel nutritional, pre-, pro- and post-biotic interventions,^110,111^ which will enable us to modulate disease phenotype through manipulation of the gut microbiota using a personalized approach.^110^
